# Response Time as a Proxy for Decision Confidence: Insights From Type-2 ROC Analysis

**DOI:** 10.1162/OPMI.a.352

**Published:** 2026-05-29

**Authors:** Kiyofumi Miyoshi, Dobromir Rahnev, Hakwan Lau

**Affiliations:** Graduate School of Informatics, Kyoto University, Kyoto, Japan; School of Psychology, Georgia Institute of Technology, Atlanta, GA, USA; Center for Neuroscience Imaging Research, Institute for Basic Science, Sungkyunkwan University, Suwon, South Korea; Department of Biomedical Engineering, Sungkyunkwan University, Suwon, South Korea; Department of Intelligent Precision Healthcare Convergence, Sungkyunkwan University, Suwon, South Korea

**Keywords:** perceptual decision-making, confidence, metacognition, response time, signal detection theory, receiver operating characteristic (ROC)

## Abstract

This study explores the use of response time (RT) data in type-2 receiver operating characteristic (ROC) analysis, a method traditionally used to examine the relationship between confidence and response correctness. Analyses of 16 perceptual decision-making datasets revealed that: (1) RT data contains roughly two-thirds as much information about response correctness as confidence; (2) RT carries unique predictive power for response correctness, independent of confidence; (3) RT and confidence interact synergistically, with confidence becoming a stronger predictor of response correctness when RT is short; (4) Despite these unique properties of RT, type-2 sensitivity (meta-d′) derived from RT and confidence showed a reasonably high correlation across subjects. These findings carry two key implications. First, in the absence of confidence data, RT can serve as a viable proxy, incurring minimal cognitive and logistical costs. This makes type-2 analysis feasible across various settings, potentially including infant and animal studies. Second, when both RT and confidence data are available, their combined use in type-2 analysis offers complementary insights into the processes underlying subjects’ behavior. To illustrate this, we present a simulation showing how the observed behavioral patterns align with the two-stage dynamic signal detection model. We propose that RT-based type-2 analysis is a valuable tool for researchers, helping to uncover previously underexplored aspects of decision-making behavior.

## INTRODUCTION

Cognitive science relies on rigorous evaluations of behavioral performance, through which researchers investigate the effects of target variables, quantify individual differences, and uncover the mechanisms underlying various psychological phenomena. Since the early days of psychophysics, receiver operating characteristics (ROCs) and signal detection theory (SDT) have been central to this purpose (Green & Swets, 1966). Due to the inherent noise in neural processing, human observers exhibit varying responses at different instances, even if exposed to identical stimuli. ROCs and SDT provide systematic frameworks for describing such probabilistic behavior, guiding principled performance evaluations.

These analytical frameworks are commonly applied when binary classification responses (hereafter referred to as primary responses) are accompanied by secondary numerical variables, such as confidence, response time (RT), or neural firing rate (e.g., Britten et al., 1992; Miyoshi & Nishida, 2024; Peters et al., 2017; Weidemann & Kahana, 2016; Wixted, 2007).^1^ Among these, confidence, along with similar introspective measures (e.g. visibility ratings, opt-out choice, or waiting time) has been employed most widely, highlighting the substantial research focus on subjective introspection within the existing research community (for a review, see Miyoshi et al., 2025).

However, when viewed solely as a secondary variable for constructing ROCs, collecting confidence ratings or related introspective measures introduces temporal and cognitive costs—an issue particularly pronounced when studying children or animals (e.g., Goupil & Kouider, 2016; Lak et al., 2014; Odegaard et al., 2018; Watanabe & Moriguchi, 2023). Therefore, the present study explores the use of RT, which does not require additional data collection and can be widely applied across various experimental situations.

**Figure 1.**
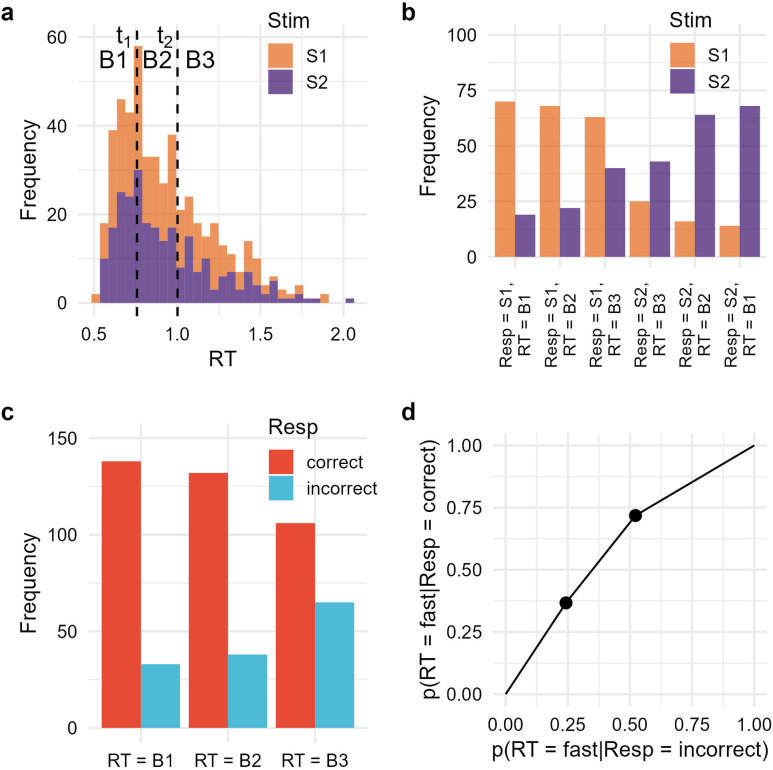
**Example of RT-based type-2 analysis.** (a) RT histogram stacked by stimulus classes (S1/S2), where observer responses (S1/S2) are aggregated without distinction. Cutoff points t_1_ and t_2_ correspond to the tertiles of the RT distribution, defining three RT bins: B1 (fastest), B2 (second fastest), and B3 (slowest). The number of RT bins can be adjusted as needed. (b) Frequency counts for categories defined jointly by stimulus class, observer response, and RT bin. These frequencies serve as the input for meta-SDT analysis. (c) The same frequency data regrouped into correct responses (stimulus and response classes match) and incorrect responses (stimulus and response classes mismatch). (d) Type-2 ROC curve showing the cumulative proportions of “fast” responses for correct and incorrect responses. The leftmost point treats only the fastest bin (B1) as “fast” and the second point considers both B1 and B2 as “fast.” The area under the curve increases to the extent that correct responses are faster than incorrect responses.

In doing so, we focused on type-2 ROC analysis, which quantifies how effectively a secondary variable discriminates an observer’s own correct and incorrect responses on a trial-by-trial basis (Figure 1; Clarke et al., 1959; Galvin et al., 2003; Maniscalco & Lau, 2012).^2^ Specifically, type-2 ROC plots the proportion of instances where the secondary variable is labeled as “high,” contingent on response correctness: p(SV = high|Resp = incorrect) and p(SV = high|Resp = correct). These proportions are plotted using varying cutoffs for the secondary variable to be considered high. The leftmost data point reflects trials exceeding the most stringent cutoff, with subsequent points calculated using progressively more lenient cutoffs. The area under the type-2 ROC curve (type-2 AUC) increases as the secondary variable becomes more diagnostic of response correctness.

Type-2 ROC analysis—and its model-based counterpart, meta-SDT analysis (e.g., Maniscalco & Lau, 2012)—has been applied almost exclusively using confidence ratings or related subjective measures, such as visibility ratings. To our knowledge, this study is the first to leverage type-2 analysis to characterize the interrelations among RT, confidence, and response correctness. Specifically, we utilized public datasets on perceptual decision-making (Rahnev et al., 2020), first conducting type-2 analyses separately for RT and confidence. We then created their composite variable and compared its type-2 diagnosticity with those of the individual variables, allowing us to investigate whether RT possesses unique information in predicting response correctness on top of confidence. These approaches would uncover behavioral patterns often overlooked in traditional analyses, offering fresh insights into the related processes.

Subsequently, to bridge findings from empirical type-2 analyses with computational modeling, we conducted a complementary simulation using the two-stage dynamic signal detection model (2DSD; Pleskac & Busemeyer, 2010) as an illustrative example. To the extent that 2DSD reproduces key empirical trends, it can inform mechanistic interpretations of the processes underlying observed data. At the same time, systematic divergences between empirical results and the model may indicate the involvement of additional computational mechanisms, including potential interplays between RT and confidence—for instance, confidence could serve as a signal to terminate information gathering (Desender et al., 2018; Lee et al., 2023).

Finally, based on observed findings, we discuss the potential of RT as a proxy for confidence. Using RT as an implicit metacognitive measure could be particularly useful in contexts where explicit introspective measures are not readily available, such as in animal studies. We, however, also outline conceptual caveats of this approach to appropriately constrain its applicability.

## METHODS

### Empirical Analyses

#### Meta-SDT Analyses.

We analyzed datasets of perceptual decision-making selected from the Confidence Database (Rahnev et al., 2020). The datasets were selected based on the following criteria: two-alternative forced-choice (2AFC) task design, availability of both RT and confidence data, and constant task difficulty (or stimulus strength) across trials within a single experimental condition (Bang et al., 2019; Rahnev & Fleming, 2019). Based on these criteria, we selected 16 datasets comprising nine different experiments and a total of 588 individuals (see Table A1 for details). Among these, 11 were excluded as they had been marked for exclusion in the original dataset, and 38 were removed due to non-convergence in the meta-SDT analysis. After additionally excluding three subjects whose d′ or meta-d′ values (see below) fell outside the range of −1 to 4, the final analysis included 536 individuals. All analyses were performed using a free statistical programming language R (version 4.3.1), with the analysis code available at https://github.com/kiyomiyoshi/rt_type2_roc.

For each dataset, we first constructed a meta-SDT analysis (Maniscalco & Lau, 2012) using confidence data. The analysis was performed at the individual level with the original maximum likelihood estimation method proposed by Maniscalco and Lau (2012), implemented via the metaSDT package. This procedure provides d′—an index of primary response accuracy—and meta-d′—an index that quantifies the diagnostic value of the secondary variable (confidence in this case) regarding primary response correctness. These two indices are directly comparable as they are both expressed in the same unit of signal-to-noise ratio (Fleming & Lau, 2014). Accordingly, we calculated meta-d′/d′ (known as m-ratio) to quantify type-2 performance relative to primary response accuracy.

Next, we performed the same analysis using RT. To maintain consistency with the confidence analysis, datasets with n-level confidence ratings had their RT data discretized into n bins, using RT quantiles as cutoffs. As with the confidence analysis, we conducted a meta-SDT analysis for each individual.

Lastly, to assess the diagnostic power of the combination of RT and confidence on response correctness, we created their composite variable using logistic regression. Specifically, logistic regression was applied for each individual to predict trial-by-trial response correctness based on RT, confidence, and their interaction. The resulting logit (log-odds ratio of response correctness) was then used as a secondary variable for meta-SDT analysis. This approach enabled a quantitative comparison of the meta-d′ index derived from the composite variable versus that obtained using confidence or RT alone. Hereafter, the meta-SDT indices calculated using the three variables above are referred to as meta-d′_confidence_, meta-d′_RT_, and meta-d′_confidence+RT_.

#### Mixed Model Analyses.

To extend the analyses above, we conducted mixed logistic regression for each dataset to predict trial-by-trial response correctness based on RT and confidence. The analysis was conducted in two stages to first evaluate the main effect of each variable and then assess their interaction. In the first stage, we specified a model with RT and confidence as fixed effects, including random intercepts and random slopes for RT and confidence across individuals. In the second stage, we added the interaction between RT and confidence as a fixed effect while retaining the same random effect structure from the first stage. While the above meta-SDT analysis summarizes observers’ performance using the standardized meta-d′ index, this mixed model approach quantifies the contribution of each variable as model coefficients and also provides formal statistical tests. The analysis was implemented in R using the lme4 package, with the statistical significance of fixed effects assessed via Type II Wald chi-square tests using the car package.

### 2DSD Simulation

#### Model Specifications.

**Figure A1.**
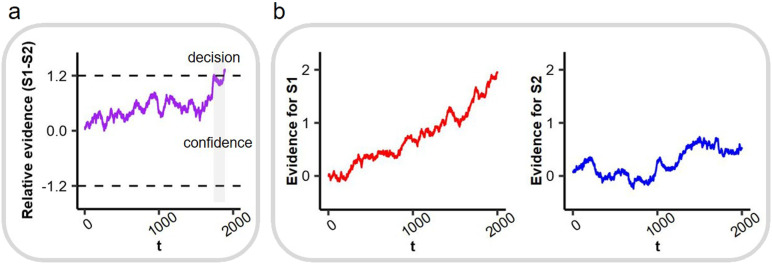
**Graphical illustration of the 2DSD model.** The 2DSD accumulator (a) was implemented by taking the difference between evidence in two independent accumulators (b). A decision is made when this relative evidence reaches either the upper or lower boundary. Confidence is then computed during the post-decisional period, highlighted in grey shading. Note that assuming independent race-to-boundary processes for the separate accumulators yields Vickers’ race model, in which confidence is determined by the difference in evidence between the two accumulators at the time of decision.

To illustrate the connection between the empirical analyses above and computational modeling, we conducted a simulation using the 2DSD model (Figure A1; Pleskac & Busemeyer, 2010). This model extends the classic drift-diffusion model (Ratcliff, 1978) by incorporating post-decisional confidence construction (e.g., Moran et al., 2015; Navajas et al., 2016).

Evidence for each stimulus (S1 and S2) was assumed to evolve over discrete time steps t (each corresponding to 1 ms), with the average rate of evidence accumulation governed by the drift rate parameter. In 2DSD, a single accumulator encodes the difference in evidence between S1 and S2, and a decision occurs when the accumulated evidence reaches either the upper or lower boundary, corresponding to a choice of S1 or S2.

To introduce meaningful trial-by-trial variability in confidence, 2DSD further assumes an additional evidence accumulation period following the decision (Fleming & Daw, 2017; Herregods et al., 2023; Miyoshi & Sakamoto, 2025; Moran et al., 2015; Navajas et al., 2016). Confidence is calculated from the difference between the evidence accumulated for chosen and unchosen stimuli during the post-decisional period (Figure A1a, grey area).

Table A2 lists the parameter values used for simulating 2DSD. The three parameters shown in bold were manipulated in the simulation. The mean target drift rate *ν*_target_ was varied as a proxy for target stimulus intensity (S1 and S2 were the correct answer an equal number of times during the simulation). Trial-to-trial variability in the drift rate (*η*) and in the accumulation starting point (s_z_) were also manipulated, given their known influence on the relationship between RT and response correctness (e.g., Ratcliff & McKoon, 2008).

#### Simulation Procedure.

We conducted the simulation using custom-built R code. The simulation was initially planned to include Vickers’ race model (Vickers, 1979), with independent decision boundaries for S1 and S2 set at a scaling value of 2. The 2DSD model was then implemented by considering the relative evidence between the two stimuli (see Figure A1 for reference). We first manually set the parameter values of *ν*_distractor_, *ν*_target_, *η*, s, s_z_, and T_er_ for the race model to cover near-chance to near-perfect performance in type-1 ROC space (see simulation code on GitHub). Across all conditions, the race model yielded an average d′ of 1.13, which guided our adjustment of the 2DSD decision boundary (a) to match this average d′. Likewise, the race model produced an average meta-d′ of 0.81, leading us to adjust the number of post-decisional confidence construction steps for 2DSD (T_pd_) to achieve a comparable average meta-d′.

For each condition, we simulated the 2DSD model over 200,000 trials, counterbalancing the target-distractor assignment between S1 and S2. In line with common practice in human experiments, we excluded trials with exceedingly slow RTs (RT > 3000) from the subsequent analyses. For confidence ratings, we used 10 quantiles of post-decisional evidence as discrete criteria to generate 10 levels of confidence (this process was carried out separately for each condition). Likewise, we discretized RTs into 10 levels using 10 quantiles, again separately for each condition. To generate the combined variable of confidence and RT, we conducted logistic regression for each condition to predict trial-wise response correctness with confidence, RT, and their interaction (confidence variables and RT were not discretized at this stage). Then, we used 10 quantiles of the resulting logits to discretize them into 10 levels. Finally, we used these three variables for meta-SDT analyses.

## RESULTS

### Meta-SDT Analyses

**Figure 2.**
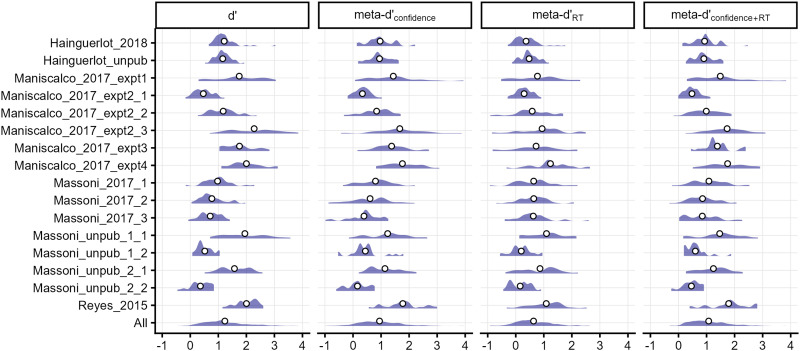
**Meta-SDT measures estimated for each dataset.** Density plots of the estimates are shown, with mean values indicated by circles. The bottom row shows results collapsed across all datasets. On average, RT carried about two-thirds of the type-2 information relative to confidence, and combining RT with confidence increased meta-d′ to roughly 1.15 times that of confidence alone.

Figure 2 presents the distributions of meta-SDT indices estimated for each dataset, with the bottom row showing results aggregated across all datasets. Across all observations (*N* = 536), the mean values were 1.23 for d′, 0.95 for meta-d′_confidence_, 0.63 for meta-d′_RT_, and 1.08 for meta-d′_confidence+RT_. That is, RT carried roughly two-thirds of type-2 diagnosticity compared to confidence, with meta-d′_confidence_ significantly higher than meta-d′_RT_ in 6 of 16 datasets (*p* < .05). Moreover, combining RT with confidence increased type-2 diagnosticity by approximately 15%, with meta-d′_confidence+RT_ significantly higher than meta-d′_confidence_ in 7 datasets (*p* < .05). Namely, while RT alone showed limited predictive power for response correctness (m-ratio = 0.5 based on the mean values above), it provided unique information beyond what confidence captured.

**Figure 3.**
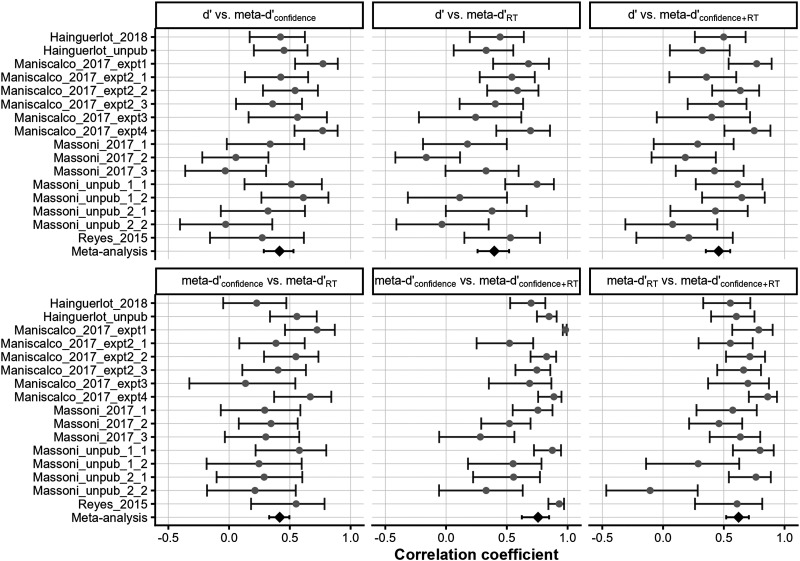
**Meta-analyses of Pearson correlations among meta-SDT measures.** Estimated *r* values are shown for each dataset, along with their 95% CIs. The bottom row displays the meta-analytic estimates integrated over all datasets. The meta-analysis revealed a moderate correlation between meta-d′ derived from RT and confidence (*r* = 0.42).

Figure 3 displays the correlations among the meta-SDT indices. Pearson’s *r* values and their 95% confidence intervals (CIs) are reported for each dataset, with the bottom row showing random-effect meta-analytic estimates across studies. Meta-d′_confidence_ and meta-d′_RT_ displayed a moderate association (*r* = 0.42, 95% CI [0.33, 0.50], *p* < .001), suggesting that meta-d′_RT_ captures meaningful individual differences in metacognitive performance. Importantly, this estimate is likely attenuated by measurement error in each meta-d′ index. To address this, we estimated disattenuated correlations using a split-half method based on odd and even trials (e.g., Eisinga et al., 2013). This analysis was restricted to 10 datasets with sufficient trials per subject and relatively high measurement reliability (Spearman-Brown coefficient > 0.45; see Table A3). Within this subset, a random-effect meta-analysis yielded a disattenuated correlation of 0.88 between meta-d′_confidence_ and meta-d′_RT_. This value was substantially larger than the meta-analytic estimate of the uncorrected correlation from the same subset (*r* = 0.51).

While these findings suggest that RT data may inform observers’ explicit metacognitive sensitivity, Figure 3 also shows that d′ correlated with all three meta-d′ indices (*p* < .001 for all). This raises the possibility that the observed correlation between meta-d′_RT_ and meta-d′_confidence_ merely reflects their shared relationship with d′. Accordingly, for meta-d′_RT_ to serve as a valid proxy for metacognitive performance, it is essential to show that its correlation with meta-d′_confidence_ remains significant after statistically controlling for primary task performance.

**Figure A2.**
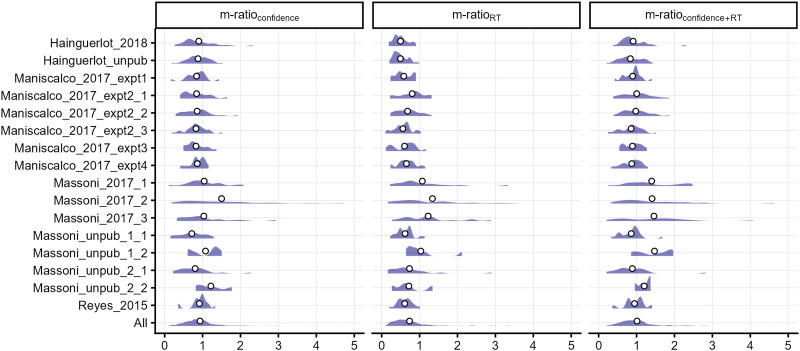
**M-ratio measures estimated for each dataset.** Density plots depict the distributions of estimates, with mean values indicated by circles. The bottom row shows results aggregated across all datasets. To avoid division by very small values, only cases with d′ and meta-d′ values between 0.2 and 4 were included in the m-ratio calculation (*N* = 343 out of 536).

**Figure A3.**
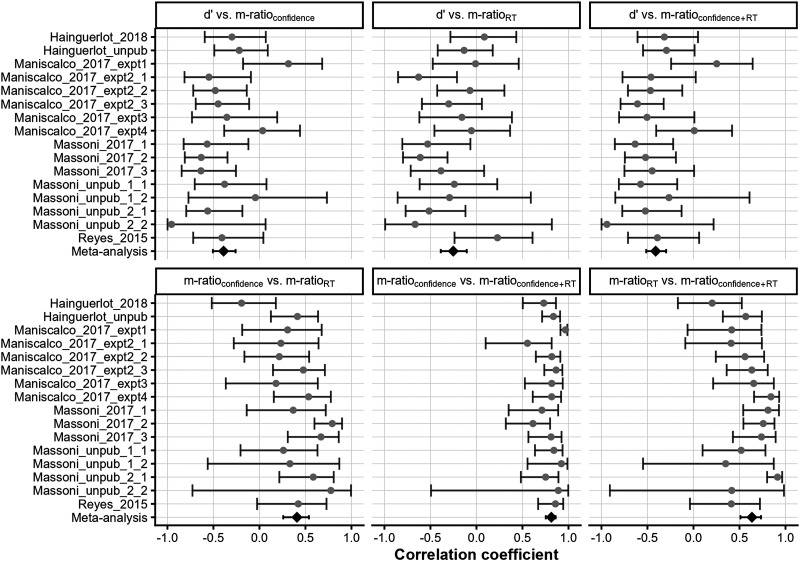
**Meta-analyses of Pearson correlations among different m-ratio measures.** Estimated *r* values are shown for each dataset, along with their 95% CIs. The bottom row displays the meta-analytic estimates integrated over all datasets, where m-ratio values derived from RT and confidence showed a moderate association (*r* = 0.43).

Figures A2 and A3 report m-ratio (meta-d′/d′) as an index serving this purpose (e.g., Fleming & Lau, 2014). A random-effect meta-analysis revealed a robust correlation between m-ratios derived from RT and confidence (*r* = 0.43, 95% CI [0.27, 0.60], *p* < .001). To supplement this finding, we additionally assessed the partial correlation between meta-d′_RT_ and meta-d′_confidence_, controlling for d′. The resulting meta-analytic estimate was *r* = 0.29 (95% CI [0.21, 0.37], *p* < .001) across datasets, representing only a modest reduction relative to the zero-order correlation reported above (*r* = 0.42, 95% CI [0.33, 0.50]). For context, a previous meta-analysis on perceptual decision-making datasets reported a correlation of *r* = 0.13 between mean accuracy and mean confidence across subjects (Jin et al., 2022). By comparison, the association observed between meta-d′_RT_ and meta-d′_confidence_ can be considered fairly strong. Together, these results indicate that meta-d′_RT_ tracks individual differences in metacognitive performance beyond variance attributable to primary task performance. Nonetheless, potential limitations of using RT as an implicit metacognitive measure are considered further in the Discussion.

### Mixed Model Analyses

**Figure 4.**
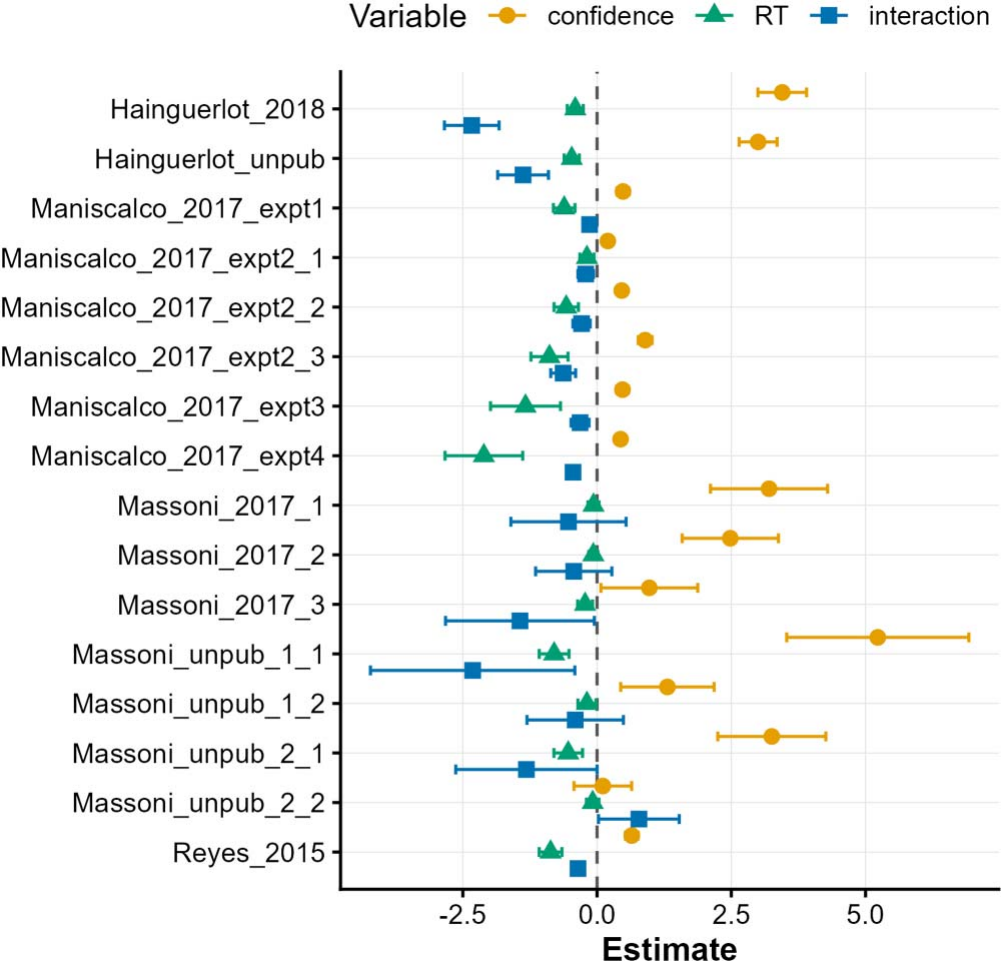
**Mixed logistic regression parameter estimates.** Symbols denote maximum likelihood estimates of the regression coefficients, and error bars indicate 95% CIs. The main effects of RT and confidence on trial-by-trial response correctness were evaluated in the first-stage estimation without including the interaction. The second-stage estimation then assessed their interaction (see Methods for details). Due to this two-stage estimation, 95% CIs that do not include zero indicate statistical significance. Overall, both shorter RT and higher confidence uniquely predicted greater response accuracy. Additionally, these variables interacted, where confidence better predicted response correctness in trials with faster RTs.

Figure 4 shows the estimated coefficients and their 95% CIs from mixed logistic regressions predicting trial-by-trial response correctness using both confidence and RT (as well as their interaction). The significance of each variable can be examined from the overlap between the CIs and the dashed line representing zero. Across the 16 datasets, RT’s contribution was significant in 13 cases, while confidence showed significance in 14 cases (*p* < .05). These findings indicate that RT independently contributes to predicting response correctness, even after controlling for the confounding effect of confidence.

Furthermore, a significant interaction (*p* < .05) was observed in 12 cases, featuring negative coefficient values, while confidence and RT had positive and negative coefficients, respectively. Given the signs of these coefficients (and insights from additional analyses below), the observed interaction can be interpreted as: (1) confidence more effectively predicted accuracy in trials with shorter RT, and (2) RT less effectively predicted accuracy in trials with lower confidence. The observed main effect and interaction involving RT were reflected in the above meta-SDT analysis, which revealed a 15% increase in meta-d′_confidence+RT_ compared to meta-d′_confidence_.

**Figure 5.**
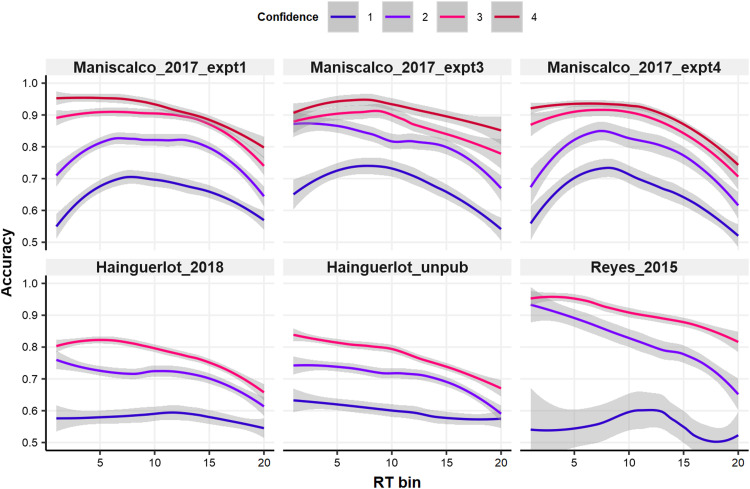
**Relationship between confidence, RT, and response accuracy.** For six datasets, response accuracy was locally smoothed across RT bins at each confidence level (shaded areas indicate corresponding *SE*s). RT bins were defined individually, with smaller values indicating faster RTs. Overall, confidence better predicted response accuracy at faster RTs. This confidence-RT interaction is reflected in variations in line slopes across different confidence levels and in inverted U patterns at low confidence levels.

To further explore the observed interaction, we selected six datasets with a relatively large number of trials per individual (see Table A1) that showed a significant interaction in the mixed modeling. First, we divided RT into 20 equally spaced bins for each subject to enhance comparability across individuals and datasets with varying RT distributions. We then plotted the average response accuracy across subjects, smoothed over different RT bins for each confidence level (Figure 5). Here, we applied locally estimated scatterplot smoothing (loess) using the default parameters of the ggplot2 package (span = 0.75, degree = 2). For estimation stability, in datasets with six confidence levels (bottom row of Figure 5), we collapsed them into three by grouping levels 1–2, 3–4, and 5–6.

On one end, the interaction is marked by steeper line slopes at higher confidence levels. This is likely because faster RTs are associated with more robust internal evidence development, leading to more efficient metacognitive monitoring (as demonstrated in later 2DSD simulations). Another key observation is that, at lower confidence levels, accuracy peaked at mid-range RT bins before declining toward the fastest RT bin, which limits the predictive value of RT for response accuracy at lower confidence levels. This inverted U pattern contrasts with the commonly assumed trend that faster RTs indicate higher accuracy. Perhaps, low confidence ratings in the fastest RT range partly reflect explicit error detection (Rabbitt, 1966; van den Berg et al., 2016; Yeung & Summerfield, 2012). That is, noisy evidence accumulation can sometimes produce extremely fast incorrect responses, but observers may consciously recognize these errors and report low confidence. This may underlie the inverted U curves observed at lower confidence levels (for further mechanistic insights, see simulation results in the next section).

### 2DSD Simulation

**Figure 6.**
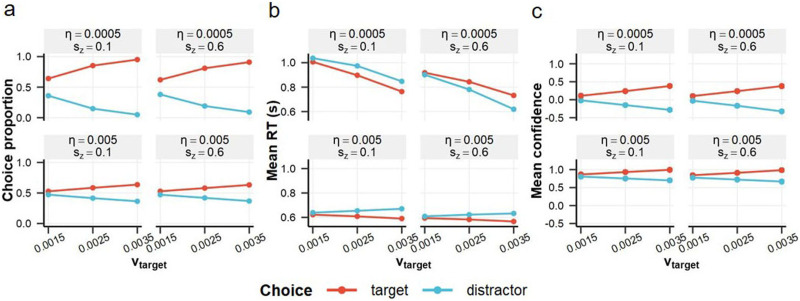
**Simulated 2DSD behavior.** (a) Target and distractor choice proportions. (b) Mean RTs for target and distractor choices. The 2DSD model exhibited flexible RT type-2 performance (i.e., relative speed of target versus distractor choices), depending on the variability in the drift rate and starting point. (c) Mean confidence for target and distractor choices. The model’s confidence closely mirrored the choice proportions, producing a “folded-X” pattern.

**Figure A4.**
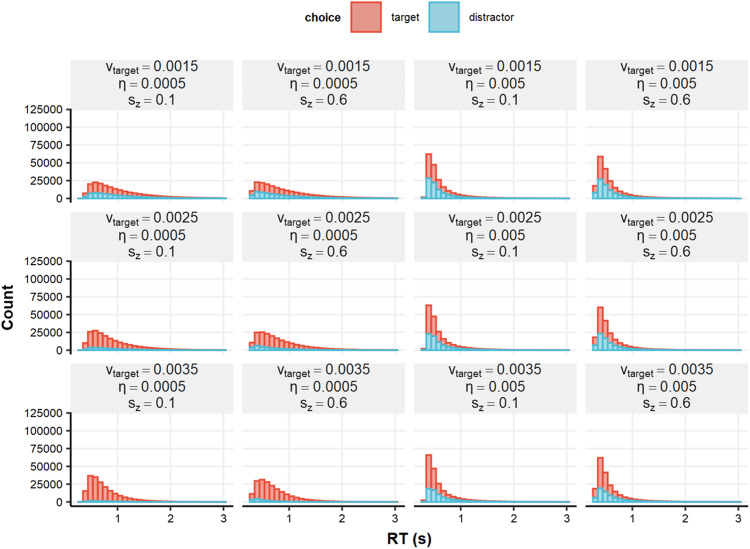
**Trial-by-trial RT distributions simulated with the 2DSD model.** Overlap between the red and blue distributions represents the type-2 diagnostic power of RT. Sequentially setting cutoffs from the faster end of the RT histograms produces RT type-2 ROCs in Figure A6.

Figure 6a shows the response proportions for each condition. The model’s decision boundary was calibrated to produce an average d′ of 1.13 across conditions. Figure 6b displays the mean RT for each condition (see Figure A4 for trial-wise RT distributions). As expected, increased drift rate variability (*η*) led to faster RTs for correct responses relative to incorrect responses (e.g., Ratcliff, 1978; Ratcliff & McKoon, 2008). That is, greater drift rate variability strengthened the association between faster RTs and correct responses, and this effect was more pronounced under higher starting point variability (s_z_).

Likewise, increased starting point variability also modulated the relative speed of correct and incorrect responses (Ratcliff & McKoon, 2008). In extreme cases, slower rather than faster RTs were associated with correct responses, demonstrating that RT can exhibit “negative” predictive power for response correctness. While relatively uncommon, this fast error phenomenon has occasionally been observed in human experiments and initially motivated the inclusion of the starting point variability parameter in the drift-diffusion model (Laming, 1968; Ratcliff & McKoon, 2008). Therefore, it is important to recognize that RT can show either a positive or negative relationship with response correctness. For confidence, negative type-2 performance is usually attributed to random measurement errors, but for RT, it can arise from the proper drift-diffusion process.

**Figure A5.**
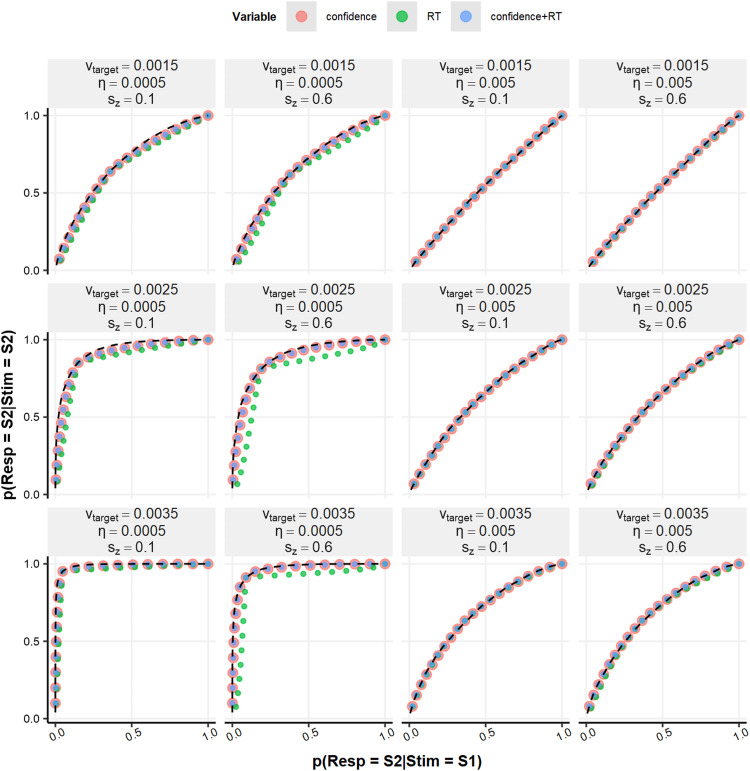
**Type-1 ROCs simulated with the 2DSD model.** Type-1 ROC analysis assesses discrimination between external stimulus states (S1 and S2) by incorporating both the primary response and a secondary variable. In this figure, type-1 ROCs are constructed by plotting the proportions of “S2” responses using varying cutoffs on the secondary variable. The leftmost point represents “S2” responses at the most stringent cutoff, with subsequent points defined with progressively more lenient cutoff values. The overall shape of type-1 ROC is largely determined by the intermediate point, which reflects the primary response alone (10th point from the left, as the simulation used 10 levels of secondary variables). The contribution of the secondary variable is captured in the other points, though its effect is primarily limited to modifying the overall ROC curvature. Consequently, differences among the three variables are relatively minor in the type-1 ROC space.

**Figure A6.**
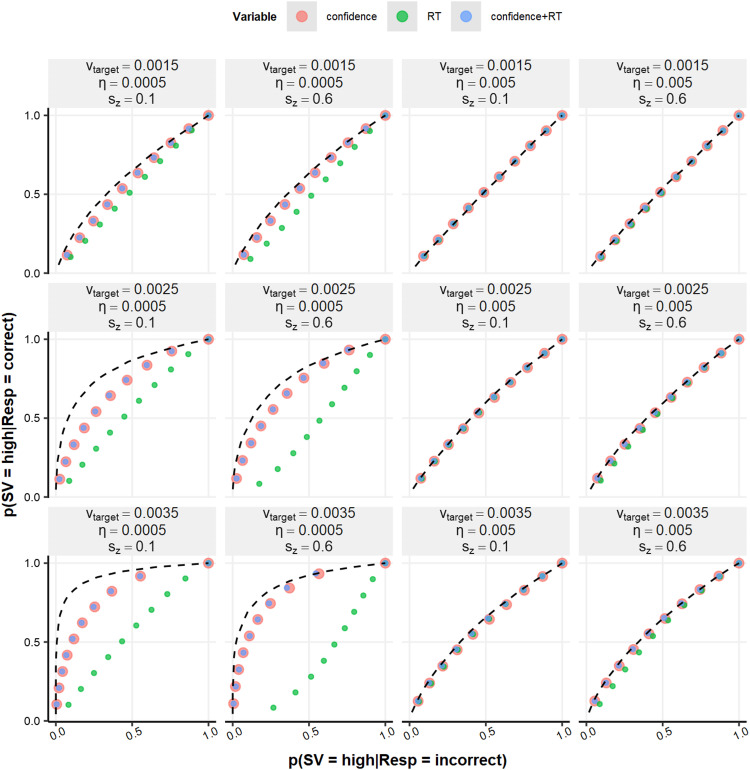
**Type-2 ROCs simulated with the 2DSD model.** Type-2 ROC analysis visualizes how a secondary variable discriminates trial-by-trial response correctness. Differences between the three variables are more pronounced in type-2 than type-1 ROC space, offering a nuanced characterization of the observer’s behavior.

Figure 6c shows the mean confidence for each condition. The 2DSD model exhibited a “folded-X” pattern, with confidence increasing for correct responses and decreasing for incorrect responses as the target drift rate increased (Miyoshi & Sakamoto, 2025; Rausch & Zehetleitner, 2019; Sanders et al., 2016; Shekhar & Rahnev, 2024). These model behaviors are further characterized by the ROC curves shown in Figures A5 and A6.

**Figure 7.**
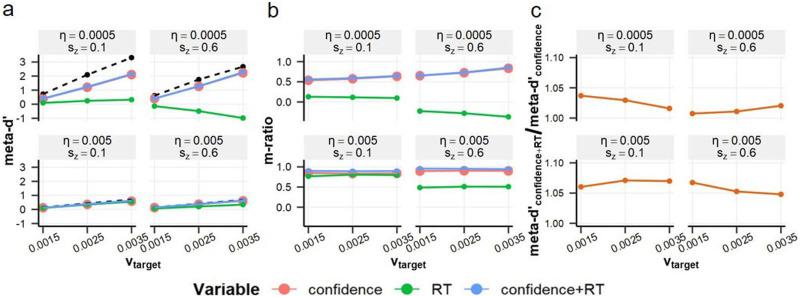
**Meta-SDT analyses on simulated 2DSD behavior.** (a) Meta-d′ estimates derived from three different variables, with dashed lines indicating d′ estimates. (b) Estimated m-ratio values. The 2DSD model produced a broad range of RT type-2 performance, aligning with the variability observed in human data. Negative m-ratio values are not meaningfully interpretable but are shown for transparency. (c) Ratio of meta-d′_confidence+RT_ to meta-d′_confidence_. The 2DSD model showed up to 1.08-fold increase in meta-d′ when combining confidence and RT, slightly below the human average of 1.15. These results highlight the 2DSD model’s ability to capture key aspects of human data, albeit with notable limitations.

Figures 7a and 7b display d′, meta-d′, and m-ratio indices estimated from the 2DSD behavior. Across parameter settings, 2DSD produced meta-d′_RT_ values that encompass the human average of approximately 0.5 and extend into negative ranges. These negative values correspond to the fast error phenomenon noted above (e.g., Ratcliff & McKoon, 2008) and were also present in empirical data (Figure 2). Figure 7c illustrates the extent to which meta-d′_confidence+RT_ exceeded meta-d′_confidence_. Here, average meta-d′_confidence_ across conditions was adjusted to 0.81 by tuning the T_pd_ parameter. Under these conditions, 2DSD showed up to 1.08 times increase in meta-d′ through the confidence-RT combination, which remains appreciably below the human average of 1.15.

**Figure 8.**
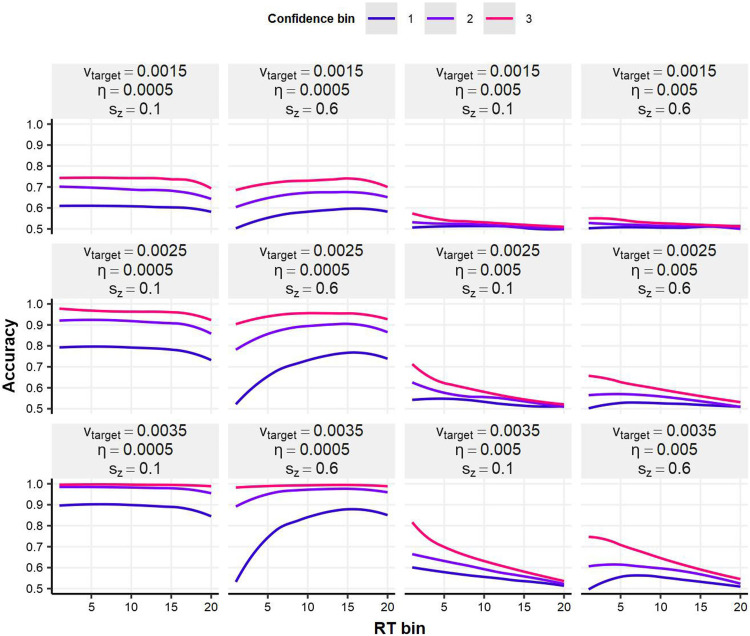
**Relationships between confidence, RT, and response accuracy in the 2DSD model.** Response accuracy was smoothed over RT bins (smaller values indicate faster responses) separately for different confidence bins (larger values represent higher confidence). In the lower-right panels, 2DSD closely replicates human data in Figure 5, showing the confidence-RT interaction (i.e., steeper slopes at higher confidence levels and inverted U curves at lower confidence levels).

Figure 8 illustrates the relationship between confidence (binned into three levels), RT (binned into 20 levels), and response accuracy. Response accuracy was calculated using local smoothing across RT bins, separately for each confidence bin. 2DSD showed a close resemblance to human data (Figure 5), demonstrating a marked confidence-RT interaction. Specifically, confidence was a better predictor of response accuracy at faster RTs, evident in steeper line slopes for higher confidence bins and inverted U curves for lower confidence bins.

Under 2DSD, faster RTs are linked to higher evidence accumulation rates, which facilitate more accurate metacognitive monitoring during the post-decisional period. This mechanism likely accounts for the slope variations observed in Figure 5. Furthermore, 2DSD revealed inverted U curves at low confidence bins, particularly under conditions of high mean target drift rate and substantial starting point variability (bottom right of Figure 8). In these scenarios, biased accumulation starting points can occasionally produce incorrect responses with short RTs. However, during the post-decisional period, the high target drift rate drives the confidence variable in a direction opposing the initial choice, resulting in low confidence ratings (i.e., high confidence in alternative choice). This can be interpreted as a form of error detection (e.g., Fleming & Daw, 2017), with the current 2DSD simulation shedding light on the dynamics driving the inverted U pattern.

Taken together, despite its simple structure, 2DSD reproduced several key empirical patterns reasonably well. This lends support to its use as a mechanistic reference for interpreting human RT type-2 characteristics.

## DISCUSSION

This study highlights the often-overlooked methodology of RT-based ROC analysis. Unlike approaches relying on confidence ratings or related subjective measures (e.g., Lak et al., 2014; Odegaard et al., 2018; Wixted, 2007), RT-based ROC analysis imposes no additional cognitive or temporal demands on observers. Moreover, it offers greater experimental ease compared to methods involving biological markers such as neural firing rates (e.g., Britten et al., 1992). Its broad applicability, including studies with animals and infants, positions RT-based analysis as a promising tool for advancing cognitive science.

The present study focused on type-2 ROC analysis, which examines the diagnostic power of a secondary variable on trial-by-trial correctness of the primary response (Figure 1). Type-2 analysis has traditionally been grounded in confidence, which has made it central to research on metacognition (Clarke et al., 1959; Galvin et al., 2003; Maniscalco & Lau, 2012; Miyoshi et al., 2025). Here, we conducted type-2 analyses using both RT and confidence to systematically quantify their relationships. Additionally, we introduced a novel method for conducting type-2 analysis based on a composite variable incorporating RT, confidence, and their interaction. As discussed below, these approaches have uncovered previously unrecognized patterns in human decision-making, providing new insights into its underlying mechanisms.

### Empirical Analyses

Our analysis of perceptual 2AFC datasets revealed that RT, on average, conveys approximately two-thirds as much information about response correctness as confidence (average meta-d′ was 0.95 for confidence and 0.63 for RT; see Figure 2). While it has long been recognized that higher confidence is typically associated with shorter RTs (e.g., Moran et al., 2015; Ratcliff & Starns, 2009; Volkmann, 1934), our study offers a formal quantification of RT’s type-2 diagnostic value relative to confidence via meta-SDT analysis.

Moreover, we showed that RT is not merely a noisier proxy for confidence but carries unique type-2 diagnosticity on its own. Specifically, the composite variable of confidence, RT, and their interaction yielded an average meta-d′ that was 1.15 times higher than using confidence alone (Figure 2). The observed interaction indicates that confidence becomes more diagnostic of response correctness at faster RTs (Figures 4 and 5), which may offer a unique window into the dynamics of perceptual decision-making.

While these findings highlight the distinct properties of RT compared to confidence, when examining the correlation across subjects, meta-d′ derived from RT and confidence showed a moderately high Pearson correlation of 0.42 in across-dataset meta-analysis (Figure 3). Correcting for attenuation due to measurement error further increased the correlation (Table A3), suggesting that RT-based meta-d′ could serve as an implicit measure of metacognitive accuracy. The feasibility of using RT as a proxy for confidence, based on this correlation, will be further discussed in the following sections.

### 2DSD Simulation

The 2DSD model (Pleskac & Busemeyer, 2010) demonstrated flexible relationships between RT and response correctness through trial-to-trial variation in drift rate and starting point (Figures 6 and 7). This flexibility allowed the model to accommodate broad meta-d′_RT_ values—including negative ones—closely mirroring the variability observed in human data.

Additionally, 2DSD replicated the confidence-RT interaction observed in human data, where confidence becomes a stronger predictor of response correctness at shorter RTs (Figure 8). In the simulation, fast RTs were associated with higher target drift rates, leading to more accurate metacognition in the post-decisional period (i.e., reduced overlap in confidence between correct and incorrect responses). The simulation further suggests that incorrect responses with short RTs—arising from starting point bias—undergo post-decisional metacognitive reevaluation, producing the inverted U pattern in the low-confidence range (Figures 5 and 8). Namely, 2DSD captured this interaction pattern through a process analogous to explicit error detection (e.g., Fleming & Daw, 2017; Rabbitt, 1966; van den Berg et al., 2016; Yeung & Summerfield, 2012).

These findings support 2DSD as a plausible basis for interpreting human RT type-2 behavior. Nonetheless, there remains room for refinement in 2DSD, as its meta-d′ improvement driven by the confidence-RT combination was smaller than that observed in humans (Figure 7c). Future research could also benefit from exploring different variations of evidence accumulation models (e.g., Ditterich, 2010; Hellmann et al., 2023; Miyoshi & Sakamoto, 2025; Rafiei et al., 2024; Usher & McClelland, 2001; Vickers, 1979), with the RT type-2 analysis proposed here providing important constraints to guide model development.

### RT as an Implicit Metacognitive Measure

The stable correlation observed between RT- and confidence-derived meta-d′ suggests that RT could serve as an implicit measure of metacognition. Notably, the correlation coefficient can be independent of the regression slope between the two variables. In other words, the correlation coefficient remains unchanged, if RT-based meta-d′ is uniformly scaled relative to confidence-based meta-d′ (e.g., by a factor of 2/3, as shown above). Therefore, while RT-based meta-d′ may not be directly comparable to confidence-based meta-d′ in absolute terms, it may still serve as a practical proxy for assessing metacognitive accuracy across individuals or experimental conditions.

Currently, our empirical findings remain correlational, and do not establish that RT directly reflects metacognitive processes. This also applies to the simulation results, since 2DSD does not explicitly define a causal relationship between RT and confidence. Instead, the 2DSD simulation suggests that RT stands in for the evidence accumulation rate and, in this indirect sense, predicts confidence levels formed during the post-decisional period. Therefore, while this study remains largely agnostic about whether RT directly reflects metacognitive processes, it highlights RT’s empirical utility as a practical proxy for confidence.

Nevertheless, numerous studies have pointed to causal links between RT and confidence. The influence of RT on confidence has long been conceptualized, as exemplified by the fluency heuristic (e.g., Ackerman & Zalmanov, 2012; Kelley & Lindsay, 1993; Thompson et al., 2013). Computational models have also been developed that explicitly incorporate RT in confidence computation (Hellmann et al., 2024; Kiani et al., 2014; Van Marcke et al., 2024). Conversely, confidence may also influence RT (e.g., Li et al., 2024). One compelling perspective is that confidence functions as a stopping signal for evidence accumulation, thereby influencing RT (e.g., Desender et al., 2018; Lee et al., 2023). If these extended models better capture human RT type-2 characteristics than the classic 2DSD model used in this study, they would offer process-level support for considering RT as an implicit indicator of metacognition.

### Future Directions

One promising avenue for RT-based type-2 ROC analysis is the examination of past datasets that lack confidence ratings—including numerous studies on infants or nonhuman animals. Future research could also benefit, as studies can be conducted more easily without the need to train animals to report confidence. RT-based type-2 analysis can also be utilized in animal neurophysiological studies. For example, studies have shown that chemical inactivation of the dorsal prefrontal cortex in monkeys (Miyamoto et al., 2017), as well as the pulvinar nucleus (Komura et al., 2013) and orbitofrontal cortex in rodents (Lak et al., 2014), affects subjective confidence levels or metacognitive accuracy. This raises the question of whether such inactivation similarly impairs meta-d′ derived from RT.

An important limitation, however, is that our target datasets exclusively involved adult human subjects. Compared with human adults, human infants and non-human animals might exhibit greater RT variability due to fluctuations in task engagement or increased noise at the motor execution level. Moreover, differences in task comprehension and developmental variation in cognitive abilities could introduce additional noise into RT and confidence data, leading to potential inter-individual differences in measurement reliability. Accordingly, establishing the validity of RT as a proxy for confidence across different target populations remains an important direction for future research.

When RT and confidence are both measured, their joint use in type-2 analysis imposes unique behavioral constraints, creating new opportunities for advancing models of perceptual decision-making. Notably, recent work has shown that different stimulus manipulations can produce qualitatively distinct relationships between response correctness and confidence (Xue et al., 2026). Specifically, manipulations of stimulus reliability (e.g., grating contrast) and stimulus distance from the category boundary (e.g., grating orientation relative to vertical) yielded “double-increase” and “folded-X” patterns of confidence, respectively, with RT exhibiting qualitatively similar trends. Combining investigations of these qualitative signatures with quantitative type-2 analyses may provide deeper insight into how distinct stimulus attributes are represented to guide decision-making behavior.

Another potential application is the investigation of the detailed temporal dynamics of confidence formation. While drift-diffusion models have largely focused on post-decisional confidence construction (Fleming & Daw, 2017; Navajas et al., 2016; Pleskac & Busemeyer, 2010), studies suggest that confidence begins to form before decision-making (Cai et al., 2022; Shekhar & Rahnev, 2018; Xue et al., 2023; Zylberberg et al., 2012). The integration of pre-decisional confidence formation into computational models remains in its early stages (Miyoshi & Sakamoto, 2025), and the joint use of RT and confidence in type-2 analysis could accelerate this effort. As highlighted, leveraging RT data in type-2 analysis offers a unique framework for characterizing decision-making behavior, opening fresh opportunities to foster research across multiple fronts.

## ACKNOWLEDGMENTS

The authors thank the academic community for providing the publicly available datasets that made this research possible. We also thank the anonymous reviewers for their constructive feedback.

## FUNDING INFORMATION

This work is supported by JSPS KAKENHI grant number 22K13870 and 25K00896. The funders have no role in study design, data collection and analysis, decision to publish, or preparation of the manuscript.

## AUTHOR CONTRIBUTIONS

K.M.: Conceptualization; Data curation; Formal analysis; Investigation; Methodology; Software; Validation; Visualization; Writing – original draft; Writing – review & editing. D.R.: Conceptualization; Methodology; Validation; Writing – review & editing. H.L.: Conceptualization; Methodology; Validation; Writing – review & editing.

## DATA AVAILABILITY STATEMENT

The data and codes are available from https://github.com/kiyomiyoshi/rt_type2_roc.

## Notes

^1^ An alternative approach for ROC construction involves manipulating the payoff of correct versus incorrect responses, or the base rate of stimulus presentation, to obtain primary response rates under different response criteria (e.g., Macmillan & Creelman, 2005). These methods fall outside the scope of the current paper, which focuses on the relationship between primary responses and secondary variables.^2^ This contrasts with type-1 ROC analysis, which concerns the classification of externally defined stimulus categories; see Miyoshi et al. (2026) for type-1 ROC analysis using RT.

## APPENDIX

**Table A1.** Dataset information

**Dataset**	**Subjects**	**Trial/Sub**	**Confidence scale**	**Task**	**Specification**
Hainguerlot_2018 (Hainguerlot et al., 2018)	52 (65)	512	6	Dot number discrimination	
Hainguerlot_unpub	52 (69)	512	6	Dot number discrimination	
Maniscalco_2017_expt1 (Maniscalco et al., 2017)	25 (30)	1000	4	Grating detection	
Maniscalco_2017_expt2_1	40 (41)	170	4	Grating detection	*ContrastLevel* = 1
Maniscalco_2017_expt2_2	40 (41)	170	4	Grating detection	*ContrastLevel* = 2
Maniscalco_2017_expt2_3	41 (41)	170	4	Grating detection	*ContrastLevel* = 3
Maniscalco_2017_expt3	20 (21)	1000	4	Grating detection	
Maniscalco_2017_expt4	25 (33)	1000	4	Grating detection	
Massoni_2017_1 (Massoni & Roux, 2017)	31 (31)	50	Continuous (0.5–1.0), made into 3 levels	Dot number discrimination	*Difficulty* = 1
Massoni_2017_2	52 (54)	50	Continuous (0.5–1.0), made into 3 levels	Dot number discrimination	*Difficulty* = 2
Massoni_2017_3	35 (35)	50	Continuous (0.5–1.0), made into 3 levels	Dot number discrimination	*Difficulty* = 3
Massoni_unpub_1_1	23 (23)	100	6	Dot number discrimination	*Study* = 1, *Difficulty* = 1
Massoni_unpub_1_2	23 (23)	100	6	Dot number discrimination	*Study* = 1, *Difficulty* = 2
Massoni_unpub_2_1	27 (27)	100	6	Dot number discrimination	*Study* = 2, *Difficulty* = 1
Massoni_unpub_2_2	27 (27)	100	6	Dot number discrimination	*Study* = 2, *Difficulty* = 2
Reyes_2015 (Reyes et al., 2015)	23 (27)	320	Continuous (1–6), made into 6 levels	Grating contrast discrimination	

The “Subjects” column indicates the number of individuals included in the present analysis, with the number before data exclusion shown in parentheses. The “Trial/Sub” column shows the number of trials per individual. For Massoni_2017, the continuous confidence range of 0.5–1 was evenly divided into three levels, while for Reyes_2015, continuous confidence ranging from 1 to 6 was rounded to the nearest integer to form six levels. The “Specification“ column indicates the conditions extracted from datasets containing multiple experimental conditions, with italicized variables representing column names in the original datasets.

**Table A2.** 2DSD model parameters

*ν*_distractor_	Mean drift rate for the distractor stimulus across trials.	0.001
***ν*_target_**	Mean drift rate for the target stimulus across trials.	0.0015, 0.0025, 0.0035
***η***	Standard deviation of across-trial Gaussian drift rate variability, applied to both target and distractor stimuli.	0.0005, 0.005
s	Standard deviation of within-trial Gaussian noise added at each time step, applied to both target and distractor stimuli.	0.03
**s_z_**	Width of the zero-centered uniform distribution representing variability in the evidence accumulation starting point for both target and distractor stimuli, with the range of ± s_z_/2.	0.2, 1.2
T_er_	Non-decision time, capturing the time required for initial stimulus encoding and motor response execution.	300
a	Decision threshold for the evidence accumulator with response boundaries set at ± a.	1.2
T_pd_	Number of time steps used for post-decisional confidence construction.	150

**Bolded** parameters represent variables of interest varied across multiple levels, while parameters in regular font remain fixed in the simulation.

**Table A3.** Attenuation correction for the correlation between meta-d′_confidence_ and meta-d′_RT_

**Dataset**	**Subjects**	**Trial/Sub**	***r*_SB_ for meta-d′_confidence_**	***r*_SB_ for meta-d′_RT_**	***r*_uncorrected_**	***r*_corrected_**
Hainguerlot_2018	52	512	0.852	0.769	0.246	0.304
Hainguerlot_unpub	52	512	0.824	0.609	0.546	0.771
Maniscalco_2017_expt1	25	1000	0.947	0.963	0.817	0.855
Maniscalco_2017_expt2_1	40	170	0.132	0.135	0.379	NA
Maniscalco_2017_expt2_2	40	170	0.728	0.646	0.506	0.737
Maniscalco_2017_expt2_3	41	170	0.752	0.817	0.225	0.287
Maniscalco_2017_expt3	20	1000	0.776	0.951	0.460	0.535
Maniscalco_2017_expt4	25	1000	0.937	0.896	0.666	0.727
Massoni_2017_1	31	50	0.520	0.046	0.235	NA
Massoni_2017_2	52	50	0.415	−0.039	0.413	NA
Massoni_2017_3	35	50	−0.616	0.257	0.362	NA
Massoni_unpub_1_1	23	100	0.348	0.185	0.578	NA
Massoni_unpub_1_2	23	100	0.467	0.480	0.246	0.519
Massoni_unpub_2_1	27	100	0.740	0.508	0.329	0.537
Massoni_unpub_2_2	27	100	0.239	0.084	0.157	NA
Reyes_2015	23	320	0.854	0.706	0.792	1

The fourth and fifth columns report reliability coefficients estimated from odd–even trials using the Spearman–Brown formula. Correction for attenuation was applied only to datasets in which both coefficients exceeded 0.45. The sixth and seventh columns present the correlation coefficients before and after this correction, respectively.
